# Reducing Loneliness and Improving Social Support among Older Adults through Different Modalities of Personal Voice Assistants

**DOI:** 10.3390/geriatrics9020022

**Published:** 2024-02-22

**Authors:** Valerie K. Jones, Changmin Yan, Marcia Y. Shade, Julie Blaskewicz Boron, Zhengxu Yan, Hyeon Jung Heselton, Kate Johnson, Victoria Dube

**Affiliations:** 1 College of Journalism and Mass Communications, University of Nebraska-Lincoln, Lincoln, NE 68588, USA; cyan3@unl.edu; 2 College of Nursing, University of Nebraska Medical Center, Omaha, NE 68198, USA; marcia.shade@unmc.edu; 3 Department of Gerontology, University of Nebraska-Omaha, Omaha, NE 68182, USA; jboron@unomaha.edu (J.B.B.); hyeonjungkim@unomaha.edu (H.J.H.); vdube@unomaha.edu (V.D.); 4 College of Computing, Data Science, and Society, University of California-Berkeley, Berkeley, CA 94720, USA; jason.yan@berkeley.edu; 5 College of Law, University of Nebraska-Lincoln, Lincoln, NE 68588, USA; kjohnson158@huskers.unl.edu

**Keywords:** voice assistant, conversational agent, older adults, loneliness, social support, interactive communication technology, multimedia learning modality

## Abstract

This study examines the potential of AI-powered personal voice assistants (PVAs) in reducing loneliness and increasing social support among older adults. With the aging population rapidly expanding, innovative solutions are essential. Prior research has indicated the effectiveness of various interactive communication technologies (ICTs) in mitigating loneliness, but studies focusing on PVAs, particularly considering their modality (audio vs. video), are limited. This research aims to fill this gap by evaluating how voice assistants, in both audio and video formats, influence perceived loneliness and social support. This study examined the impact of voice assistant technology (VAT) interventions, both audio-based (A-VAT) and video-based (V-VAT), on perceived loneliness and social support among 34 older adults living alone. Over three months, participants engaged with Amazon Alexa™ PVA through daily routines for at least 30 min. Using a hybrid natural language processing framework, interactions were analyzed. The results showed reductions in loneliness (Z = −2.99, *p* < 0.01; pre-study loneliness mean = 1.85, SD = 0.61; post-study loneliness mean = 1.65, SD = 0.57), increases in social support post intervention (Z = −2.23, *p* < 0.05; pre-study social support mean = 5.44, SD = 1.05; post-study loneliness mean = 5.65, SD = 1.20), and a correlation between increased social support and loneliness reduction when the two conditions are combined (ρ = −0.39, *p* < 0.05). In addition, V-VAT was more effective than A-VAT in reducing loneliness (U = 85.50, *p* < 0.05) and increasing social support (U = 95, *p* < 0.05). However, no significant correlation between changes in perceived social support and changes in perceived loneliness was observed in either intervention condition (V-VAT condition: ρ = −0.24, *p* = 0.37; A-VAT condition: ρ = −0.46, *p* = 0.06). This study’s findings could significantly contribute to developing targeted interventions for improving the well-being of aging adults, addressing a critical global issue.

## 1. Introduction

The population of those over 60 years old will double by 2050, and the population of those over 80 is expected to triple [1]. Healthy aging is a global priority. Older adults, particularly those living alone, report higher perceived loneliness and lower perceived social support, which are associated with numerous physical and mental health outcomes [2]. In fact, higher health care utilization, blood pressure, depression, and anxiety are associated with loneliness and low social support [3,4].

A variety of interventions using interactive communication technologies (ICTs) may help reduce feelings of loneliness and increase social support. Communication via email, smartphones, iPads, chat rooms or forums, the Wii, and virtual pet companions have all been reported to positively influence loneliness [5]. Unfortunately, the usability of many ICTs can pose as a barrier for older adults due to declines in eyesight, dexterity, and cognition [6]. 

Prior studies about the use of voice assistants as an intervention for loneliness and social support among older adults are limited in scope. Those that exist largely focus on acceptability, user experience, satisfaction, usability, or performance [7,8,9]. Intervention studies have focused on forming connections with strangers and not within the older adult’s previously formed relationships [10]. We were unable to locate existing studies that have compared the modality of voice assistant artificial intelligence (AI) as an intervention, and how video or audio interaction may influence outcomes. The purpose of this study was to explore if and how loneliness can be reduced and social support increased among aging adults using AI-powered personal voice assistants (PVAs) through different modalities. 

### 1.1. Loneliness and Social Support in Older Adults

The experience of loneliness is one of the most influential factors in quality of life among aging adults, affecting physical health, mental health, and emotional well-being [11,12]. Loneliness is the subjective feeling of being alone, lacking companionship, or not belonging [13], and has been associated with higher rates of depression, self-harm, and self-neglecting behavior [14,15]. It has also been shown to predict functional decline and death [13,16]. In fact, during the COVID-19 pandemic, limited human contact caused feelings of loneliness to increase considerably, particularly among older people [17,18].

Social support refers to the social ties to others—individuals, groups, or communities—who can provide support to an individual [19,20]. This could include the perception that support would be available if needed (perceived social support) or the actual receipt of help (received social support) [21]. Social support tends to diminish with age; social networks get smaller with retirement, the loss of family and friends, changes in economic status, and physical or cognitive limitations that contribute to reduced activity, connection, and engagement [4,22,23]. Meta-analysis data indicate a significant relationship between perceived social support and reduced depression and anxiety [24]. 

There is a relationship between loneliness and social support. Individuals with higher levels of social support have been reported to report lower levels of loneliness [25]. Also, less social support and greater social isolation are associated with greater loneliness [26]. Perceived social support has been shown to have more of an effect on loneliness than received social support [27].

### 1.2. Information and Communication Technologies and Modality

A variety of information and communication technologies (ICTs) have been used to help mitigate feelings of loneliness among aging adults and provide social support. ICTs include personal voice assistants (PVAs) such as Alexa [28,29], also known as conversational voice assistants (CVAs), voice-controlled intelligent virtual or personal assistants (VIPAs or IPAs), smart speakers, smart assistant technology (SAT), smart voice assistants, or voice-activated virtual home assistants (VHAs). These technologies are powered by artificial intelligence and respond to the human voice [30,31,32,33]. PVAs have been shown to reduce perceptions of loneliness and provide older adults with a sense of companionship [28,31,34]. An analysis of Alexa use among older adults in long-term care suggest that nearly 22% of the interactions with Alexa relate to well-being, as residents get to know Alexa, ask for advice, and call or message others [35]. Higher levels of internet use among older adults can predict higher levels of social support, reduced feelings of loneliness, and better psychological well-being and life satisfaction [36]. Thus, internet-based interactive communication technologies (ICTs) may increase perceived social support and decrease loneliness [24,26]. 

While a PVA device is operated by voice, the embedded artificial intelligence-powered system can interact with the user through either audio or video. These different modalities may influence how loneliness and social support are affected. Research indicates that learning from video that combines visuospatial and auditory information is better than learning from audio with auditory information only [37]. Mayer and Moreno’s [38] cognitive theory of multimedia learning (CTML) posits that combining visual and auditory information can facilitate deeper learning by engaging dual channels of the brain, thus optimizing cognitive processing. This theoretical framework is anchored in the foundational principles of cognitive load theory and information processing theory [39]. In the CTML framework, cognitive load is conceptualized as a state that emerges when irrelevant stimuli compete for the limited processing bandwidth of working memory, thereby hindering effective learning. On the other hand, information processing in the CTML model delineates the trajectory of information acquisition, positing that acquired information undergoes a sequential transition through various memory stages, facilitated by a constellation of cognitive processes, culminating in its consolidation in long-term memory. Mayer and Moreno [39] argue that the utilization of multimedia in learning contexts necessitates the optimization of cognitive load to enhance the efficiency of information processing within the memory system. 

Considering that cognitive processes such as memory, attention, and the speed of processing undergo changes with aging [40], studies have shown that older adults benefit from multimedia learning principles by managing cognitive load [41] and enhancing their engagement with technology [42]. Older people’s video-based learning via both the phonological loop and the visuospatial sketch pad likely produces better comprehension and recall and stronger self-reported engagement, whereas audio-only learning tends to over-tax the phonological loop, causing selective working memory interference [37]. Interventions using multimedia learning principles have shown promise in reducing barriers to technology adoption among older people, including anxiety, a lack of familiarity, and perceived complexity [43]. As such, for aging adults, video-based PVA devices seem to have an advantage over audio-based PVAs.

Research on PVA-based interventions is still emerging and limited to testing if the technology can help manage loneliness among older adults, while how it produces such outcomes via the modality effect has not been explored. The purpose of this study was to examine a novel intervention using different modalities (i.e., audio vs. video) of PVA-based multimedia interaction with commercially available voice-powered AI assistants, such as Amazon Alexa (Echo Dot as the audio-only PVA (A-PVA) or Echo Show as the video PVA (V-PVA)), to address loneliness and social support among older people. The following hypotheses were therefore proposed.

**H1:**  *Voice assistant technology-based (VAT) interventions including both video-based VAT (V-SAT) and audio-based VAT (A-VAT) will reduce perceived loneliness among older adults living alone after three months of regular use.*

**H2:**  *VAT interventions including both video-based VAT (V-VAT) and audio-based VAT (A-VAT) will increase perceived social support among older adults living alone after three months of regular use.*

**H3:**  *Perceived social support will reduce perceived loneliness among older adults living alone after three months of regular use.*

**H4:**  *Participants in the V-PVA intervention will report significantly stronger reductions in perceived loneliness than those in the A-PVA intervention after three months of regular use.*

**H5:**  *Participants in the V-PVA intervention will report significantly stronger increases in perceived social support than those in the A-PVA intervention after three months of regular use.*

**H6:**  *The effect of perceived social support on reducing perceived loneliness among older adults living alone will be stronger in the V-PVA intervention than the A-PVA intervention after three months of regular use.*

## 2. Method

To be eligible for the study, participants needed to be fluent in English, live alone in an independent living facility in the United States, be aged 50 or more, and have used an Amazon Echo or Google Nest an average of zero to three times per week in the past 30 days. They also needed to be willing to use an Amazon Alexa™ PVA for 30 min or more, per day, for 12 weeks, and complete an assessment of capacity to consent, administered by the researchers. Participants (*n* = 34; 38% male, 62% female; mean age = 77, SD = 11.52, age range: 50–98; 94% White, 3% African American, and 3% Asian American) engaged in predetermined daily routines composed of activities from prior studies meant to help address loneliness as well as activities that were commonly engaged in through PVAs: greetings (Good Morning, Good Afternoon and Evening, Goodnight), Big Sky, Daily Riddle, Five Minute Morning Meditation, Music, Weather, Asking for a Joke, Playing the Akinator Guessing Game, and Calls to existing social connections. Instructions provided to participants outlined which of these activities to carry out through Alexa at what time, divided between morning, afternoon, and evening. These instructions are located in the Supplementary Material.

Prior to the commencement of the study, participants underwent the Montreal Cognitive Assessment (MoCA) [44], yielding an average MoCA score of 23.47 (SD = 3.65, score range: 14–30). To avoid intervention interference at the same recruitment site, a cluster randomized trial method was used to assign treatment conditions (A-PVA: Amazon Echo Dot; V-PVA: Amazon Echo Show). Participants from the same independent living facility were assigned to the same treatment group. This project was approved by the University of Nebraska-Lincoln Institutional Review Board, #20220321416FB, and all participants signed an informed consent form.

### 2.1. Measurement

Each participant’s perceptions of loneliness and social support were measured immediately before the study (baseline), and after 12 weeks (week 12) of use. A data log that recorded all participant voice interactions with the PVA during the entire 12 weeks was created for each participant.

### 2.2. Loneliness

Loneliness was measured using the UCLA loneliness scale [45] immediately before the intervention and after 12 weeks during which participants were required to complete daily routines on the PVA. The 20 items were assessed on a 4-point scale ranging from one (I never feel this way) to four (I often feel this way). A summary perceived loneliness score was calculated for baseline and week 12, respectively. Cronbach’s alpha was 0.95 for baseline loneliness and 0.95 for week 12 loneliness. Post-study loneliness was subtracted from baseline loneliness to create a loneliness change score for each participant.

### 2.3. Social Support

Perceived social support was measured using the multidimensional scale of perceived social support (MSPSS) [46]. The MSPSS was designed to measure perceived social support and is widely used for testing social support’s relationship with depression and anxiety among older adults [47]. The 12 items were assessed on a 7-point scale ranging from one (strongly disagree) to seven (strongly agree). Cronbach’s alpha was 0.92 for baseline perceived social support and 0.95 for week 12 perceived social support. Post-study social support was subtracted from baseline social support to create a social support change score for each participant.

### 2.4. Coding of Older People’s Voice Interactions with PVA

Researchers often use dictionary-based text analysis to study older adults’ interactions with PVA and its impacts, focusing on keywords to understand user behavior and ethical AI considerations [28,48,49]. However, this method oversimplifies interactions as it does not capture the two-way communicative nature of these exchanges or the progression of conversations over time. Standard tools like the Linguistic Inquiry and Word Count (LIWC) only measure word frequencies [50], failing to recognize the dynamic and adaptive nature of SVA interactions.

Moreover, as people age, changes in cognitive abilities and speech patterns may lead to more complex PVA interactions involving repetitions, corrections, and confirmations [51,52]. To address these limitations, we developed a hybrid natural language processing (NLP) framework that combines a modified rule-based NLP model with human input (Authors, under review). This approach, which is platform-independent and replicable, blends manual and automated coding to analyze older adults’ voice interactions with PVAs. It leverages human insights to build a keyword lexicon and iteratively refine the NLP model, capturing the nuanced dialogue dynamics in older adults’ PVA use.

We developed a hybrid coding framework that combines the human ability to understand complex speech patterns of older people with the computational efficiency of NLP algorithms, leveraging the strengths of both for enhanced performance. In this hybrid framework, a panel of experts first developed a comprehensive coding schema with 12 predefined categories of daily routines and interactions to capture the prescribed 10 daily routines meant to reduce loneliness, as well as setting adjustments and other interactions outside the scope of the pre-programmed daily routines. Secondly, a human coder then coded a portion of the data to set coding standards for classifying keywords and commands into the 12 predefined PVA interaction categories. Thirdly, using the coding schema and standards developed by the expert panel and the human coder, an expert NLP programmer created NLP algorithms and developed software to automatically code the same portion of the data. Fourthly, the human coder and the NLP programmer worked together to refine and validate the NLP algorithms. Finally, the modified NLP algorithms were used to the entire data set. The methodology for creating this hybrid framework, which integrates human expertise with natural language processing models, is thoroughly documented to enable replication (Authors, under review). Additionally, an online version compatible with various computer platforms is available at https://excel-helper-deploy.vercel.app accessed 3 November 2023.

### 2.5. Coding Schema Development by a Human Coder

Participant-generated commands and corresponding Amazon Alexa™ Smart Voice Assistant (SVA) responses were systematically captured and extracted from individual Amazon SVA accounts. The final data set comprised time-stamped, textual interactions, systematically arranged in chronological order across 35 distinct Microsoft Excel files. For preliminary analysis, a stratified subset constituting 21% of the data (*n* = 7, encompassing 1020 interactions) was subjected to manual coding by a human coder. A comprehensive coding schema was devised to categorize interactions based on their relevance to the completion of daily routines and other significant exchanges. This schema utilized specific keywords and commands to classify the data into 12 predefined categories of daily routines and additional interaction types such as entertainment (including music, jokes, games/Akinator, riddles, meditation/Five Minute Morning), information acquisition (e.g., weather/Big Sky), greetings, phone calls, settings adjustments, and interactions falling outside the scope of the pre-programmed daily routines. Among the 12 categories, music was the most common activity. Utilizing the coding schema detailed in the Supplementary Material, we interpreted a participant’s voice command, “Play country music George Strait for five minutes”, as a single activation within the music category.

### 2.6. Rule-Based NLP Coding

**Figure 1:**
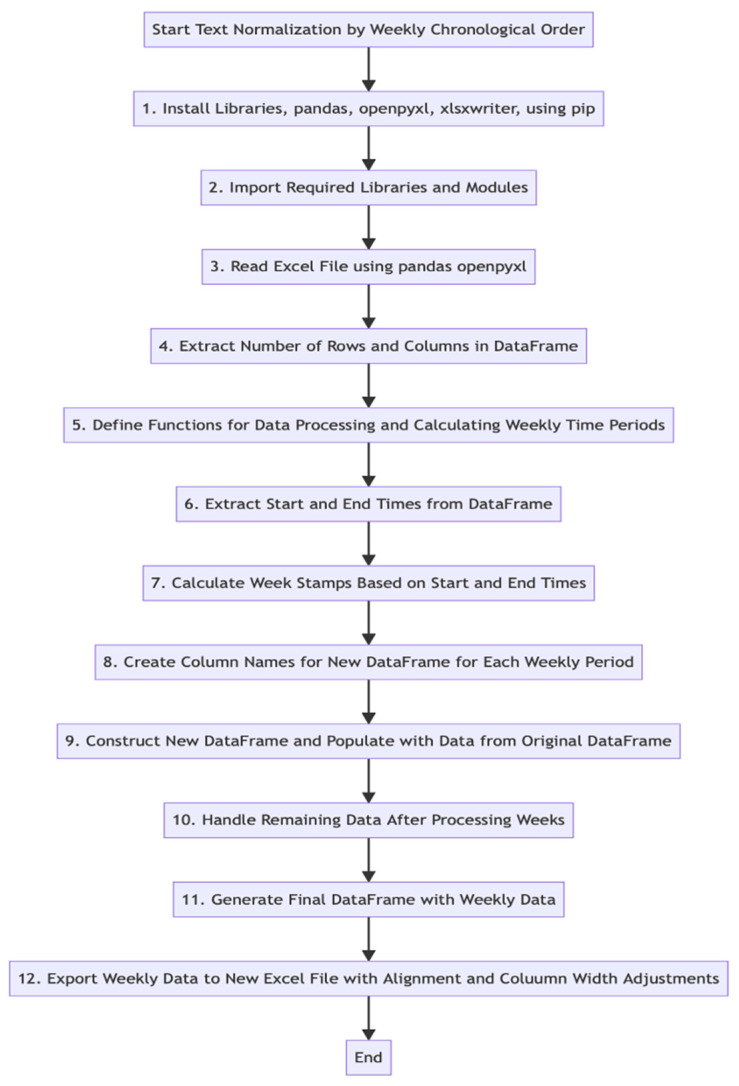
Text normalization.

An expert NLP programmer, following the NLP rule development approach [53], reviewed the coding standards set by the human coder to understand the classification of keywords and commands. Using this understanding, knowledge-based rules were applied for NLP coding of the same subset. To perform text normalization, the raw data were sorted chronologically, into weekly segments, using libraries such as pandas, openpyxl, xlsxwriter, and the Python programming language. This process was applied to the same 21% data subset (*n* = 7). The sorted weekly data were stored in a new DataFrame for enhanced visualization and analysis. The text normalization process is detailed in Figure 1, created with Mermaid v10.5.0 Live Editor.

**Figure 2:**
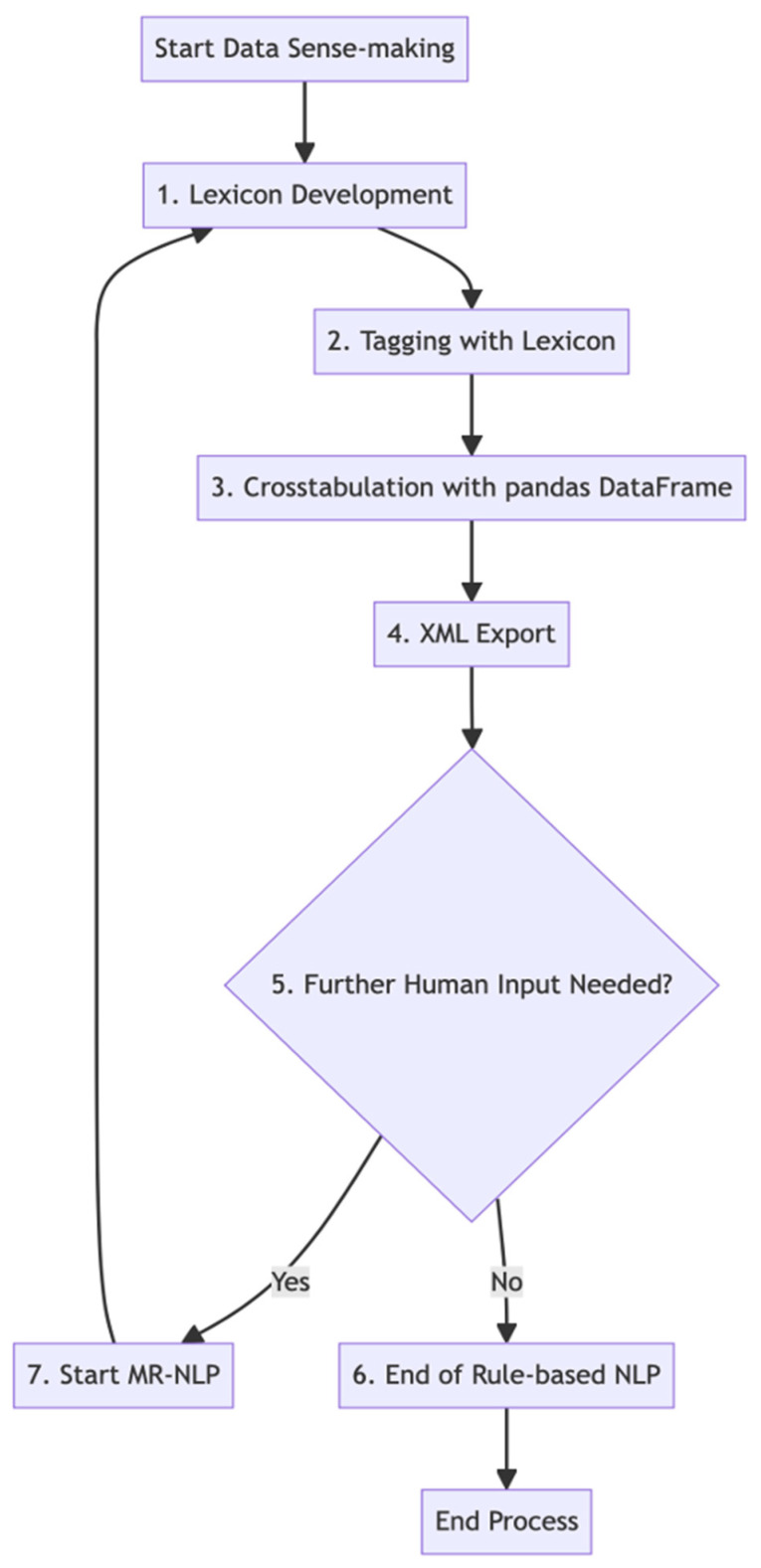
Data sense-making using rule-based NLP or modified rule-based NLP.

Leveraging the same 12 categories from manual coding, the 21% subset data set underwent processing via Python, utilizing an XML export feature and libraries like TextBlob and pandas. TextBlob was particularly effective for tasks such as word extraction and comparing user commands. The aim was to employ rule-based NLP to glean insights from user commands and responses. A set of keywords was determined, and functions were designed for keyword identification in user inputs, using text processing methods like tokenization. Commands were categorized based on these keywords, considering both single and repeated command inputs as successful routine completions. The analysis results, showing keyword frequencies across weeks, were cross-tabulated and facilitated by XML export for better data integration. The process of rule-based NLP coding across different routine categories is detailed below and depicted in Figure 2, created with Mermaid v10.5.0 Live Editor.

The human coder and NLP programmer discussed and resolved discrepancies (14.51% of the subset data, 148 cases). Based on knowledge learned from the discussion, the rule-based NLP technique was modified to code the entire data set among all 35 participants accordingly. Participants reported an adequate level of engagement with the device (mean 12-month routine completions = 714.26, SD = 448.01, averaging 8.50 daily).

## 3. Statistical Analysis

This study employed various statistical methods to analyze the effectiveness of voice assistant technology-based (VAT) interventions on perceived loneliness and social support among older adults. The analysis was segmented based on specific hypotheses.

1. General Statistical Procedures:
  - Descriptive Statistics and Normality Tests: Initially, descriptive statistics were calculated for the study’s dependent variables—perceived loneliness and perceived social support—across all intervention conditions. The Shapiro–Wilk test was utilized to assess the normality of these variables, determining the appropriateness of subsequent tests.
2. Hypotheses 1 and 2 (H1 and H2):
  - Wilcoxon Signed-Rank Test: For H1 and H2, where normality was not assumed (as indicated by the Shapiro–Wilk test), the Wilcoxon signed-rank test, a nonparametric test, was used. This test compared pre- and post-study scores for both perceived loneliness and social support among all conditions combined.
3. Hypothesis 3 (H3):
  - Spearman’s Rank Correlation Test: To explore the relationship between changes in perceived social support and perceived loneliness (H3), a two-tailed Spearman’s rank correlation test was conducted at a 95% confidence interval. This test was chosen due to its suitability for nonparametric data.
4. Hypotheses 4 and 5 (H4 and H5):
  - Mann–Whitney U Test: For comparing the effectiveness of V-VAT and A-VAT interventions on perceived loneliness and social support (H4 and H5), the Mann–Whitney U test was applied. This test is appropriate for comparing differences between two independent groups, in this case, the V-VAT and A-VAT intervention groups.
5. Hypothesis 6 (H6):
  - Spearman’s Rank Correlation Test for Subgroups: For H6, the Spearman’s rank correlation test was again employed, this time to separately examine the relationship between changes in perceived social support and loneliness in the V-VAT and A-VAT conditions. The choice of this test was due to its effectiveness in handling nonparametric data in small sample sizes.

Each of these methods was chosen based on the nature of the data and the specific requirements of each hypothesis. This comprehensive approach ensured the robust and reliable analysis of the study’s outcomes.

## 4. Results

### 4.1. Loneliness Reduction and Social Support Increase

Descriptive statistics and normality tests (see Table 1) were performed for the study’s dependent variables, i.e., perceived loneliness and perceived social support, among all intervention conditions.

**Figure 3:**
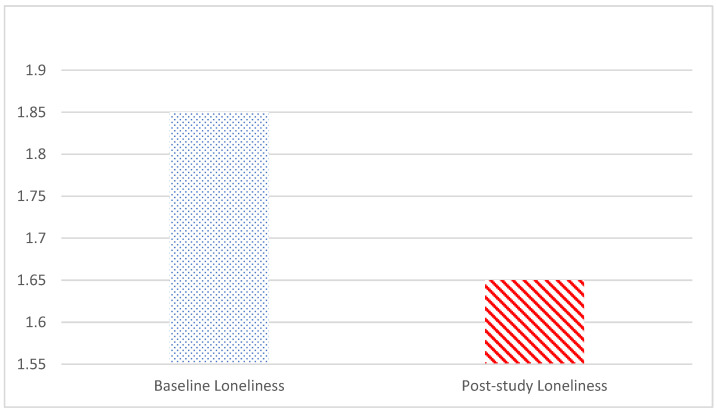
Comparison of pre- and post-study loneliness mean scores.

**Figure 4:**
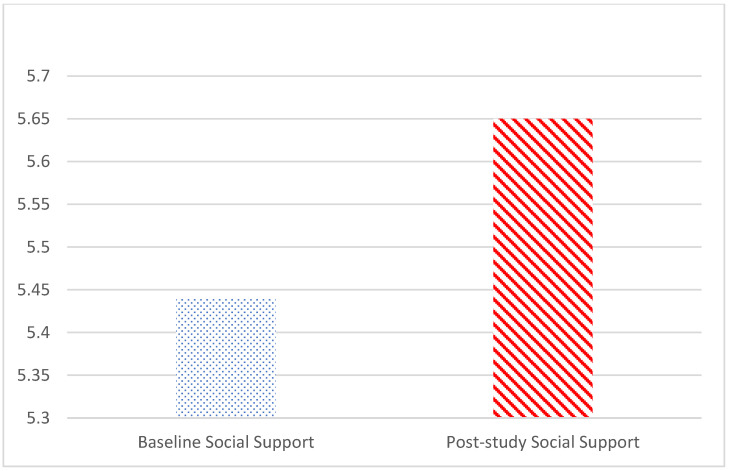
Comparison of pre- and post-study social support mean scores.

Based on the Shapiro–Wilk test, the null hypothesis of normal population distribution was rejected at α = 0.05 for post-study loneliness (W(34) = 0.86, *p* < 0.001) and post-study support (W(34) = 0.87, *p* < 0.001), while it failed to indicate a deviation from normal population distribution for baseline loneliness (W(34) = 0.96, *p* = 0.266) and baseline social support (W(34) = 0.94, *p* = 0.072). Consequently, the Wilcoxon signed-rank nonparametric test was performed to compare pre- and post-study loneliness scores and pre- and post-study social support scores. The two-tailed Wilcoxon signed-rank test showed that participants reported reductions in perceived loneliness (Z = −2.99, *p* < 0.01; pre-study loneliness mean = 1.85, SD = 0.61; post-study loneliness mean = 1.65, SD = 0.57; see Figure 3) and increases in perceived social support (Z = −2.23, *p* < 0.05; pre-study social support mean = 5.44, SD = 1.05; post-study loneliness Mean = 5.65, SD = 1.20; see Figure 4) after the intervention among all conditions combined, supporting H1 and H2. There were a significant loneliness reduction and a social support increase at the end of the intervention.

### 4.2. The Relationship between Social Support and Loneliness

A two-tailed Spearman’s rank correlation test was performed at the 95% confidence interval to examine the relationship between changes in perceived social support and changes in perceived loneliness at the end of the 12-month intervention among all conditions. The results indicated a significant correlation between perceived social support (mean = 0.46, SD = 0.77) and changes in perceived loneliness (mean = −0.27, SD = 0.39): ρ = −0.39, *p* < 0.05, *n* = 34). Therefore, H3 was supported. An increase in perceived social support indeed led to a significant reduction in loneliness perception.

### 4.3. Effects of VAT Modality

A Mann–Whitney U test was conducted to compare changes in perceived loneliness between the V-VAT (*n* = 16) intervention and the A-VAT (*n* = 18) intervention. The results indicated that there was a statistically significant difference in changes in perceived loneliness between the two groups: U = 85.50, *p* < 0.05. The median change score of perceived loneliness for the V-VAT condition was −0.45, while for A-VAT it was −0.15. Therefore, H4 was supported. Video-based VAT produced a stronger loneliness reduction than audio-based VAT.

A Mann–Whitney U test was conducted to compare changes in perceived social support between the V-VAT (*n* = 16) intervention and the A-VAT (*n* = 18) intervention. The results indicated that there was a statistically significant difference in changes in perceived social support between the two groups: U = 95, *p* < 0.05. The median change score of perceived social support for the V-VAT condition was 0.76, while for A-VAT it was 0.25. Therefore, H5 was supported. Video-based VAT produced a stronger increase in social support than audio-based VAT.

A two-tailed Spearman’s rank correlation test was performed at the 95% confidence interval to examine the relationship between changes in perceived social support and changes in perceived loneliness at the end of the 12-month intervention in the V-VAT condition and the A-VAT condition, respectively. The results failed to support a statistically significant correlation between perceived social support (V-VAT: mean = 0.76, SD = 0.80; A-VAT: Mean = 0.20, SD = 0.66) and changes in perceived loneliness (V-VAT: mean = −0.41, SD = 0.35; A-VAT: mean = −0.14, SD = 0.37) in either the V-VAT condition (ρ = −0.24, *p* = 0.37, *n* = 16) or the A-VAT condition (ρ = −0.46, *p* = 0.06, *n* = 18). Therefore, no significant correlation between changes in perceived social support and changes in perceived loneliness was observed in either intervention condition. H6 was not supported. VAT intervention modality did not make a significant impact on the relationship between social support and loneliness perception.

## 5. Discussion

People are living longer, and the population of older adults is growing at an unprecedented rate [54]. Older adults living alone are more at risk of experiencing loneliness, which can significantly influence their health and well-being [2]. This research suggests that voice-activated ICTs like PVAs can help reduce feelings of loneliness and increase social support among older adults, whereas other ICTs may be more challenging for this population to use [6]. As demonstrated, these devices can not only respond to and interact with participants, but also facilitate connections between participants and their social support networks through activities such as calling. Based on our data, video-based PVAs have a greater influence on loneliness and social support than audio-only PVAs.

These specially designed VAT interventions reduced loneliness and increased social support during the 12-week intervention. This aligns with and builds on prior findings about loneliness reduction through PVAs [28,31], adding a social support component. Activities were selected based on the existing literature to address loneliness—activating humor by asking for jokes, stimulating cognition and challenge by asking for a riddle and an interactive game, improving emotional states and stress through meditation and music, greeting the PVA with “Good morning” and “Good afternoon/evening” to personify it [28,55], and video- or audio-calling friends or family. These activities encouraged engagement with and through the PVA.

The analyses indicated that perceived social support did indeed reduce perceived loneliness among older adults living alone after three months of regular use. As noted earlier, the mix of activities participants engaged in with the PVA was intentional and unique to this study. Further, the few other studies about PVAs and loneliness did not require video or audio calls through the device. This interaction with the always-ready, responsive AI and this connection with social ties may have influenced perceptions of social support.

Another significant contribution from our research is the proposed modality effect of voice assistant technology on loneliness reduction and social support induction. As hypothesized, participants in the V-VAT intervention indeed experienced significantly stronger reductions in perceived loneliness and greater increases in perceived social support than those in the A-VAT condition. According to this theoretical paradigm, the utilization of both visual and auditory stimuli in the V-VAT learning process facilitates a more effective integration and processing of information among aging adults. In our study, participants who were enrolled in the video condition, as opposed to those in the audio-only condition, demonstrated a marked advantage in mitigating feelings of loneliness and in bolstering their perceived levels of social support. This phenomenon can be interpreted through the lens of the CTML theory, which posits that the dual channels for processing information—one for visual/pictorial material and another for auditory/verbal material—are more optimally engaged when both modalities are present.

The video condition, by virtue of presenting information through both auditory and visual channels, likely facilitated a more comprehensive cognitive processing. This dual-channel engagement potentially led to a more profound internalization of the content, which in turn could have contributed to a greater sense of connection and social support among participants. In contrast, the audio-only condition, relying solely on the auditory channel, may have resulted in a more limited cognitive engagement, thereby lessening the impact on participants’ feelings of loneliness and their perception of social support.

Furthermore, the element of social presence, which is more pronounced in video formats due to the inclusion of visual cues such as facial expressions and body language, could also play a significant role in this observed modality effect. The enhanced sense of presence and connection afforded by the video format might have contributed to the participants’ improved ability to chip away at feelings of loneliness and shore up their sense of social support.

Ultimately, all hypotheses were supported except the last one, H6. The analysis did not indicate that the effect of perceived social support on reducing perceived loneliness among older adults living alone was stronger in the V-VAT intervention than the A-VAT intervention. This could be due to the relatively small sample size. Data from our study did not reveal a statistically significant difference in the efficacy of the video-based intervention compared to the audio-only intervention with regard to the impact of perceived social support on mitigating perceived loneliness among older adults living alone. The absence of a discernible difference in outcomes between these two intervention modalities could be attributed to the limitations imposed by the relatively small sample size used in the study. In light of the significant modality effects reported in the results for all other five hypotheses, we could speculate that the small sample size could introduce a higher likelihood of Type II errors, where a true effect or difference exists but goes undetected due to the inadequate sample size. Consequently, the null findings in this study should be interpreted with caution, as they might not necessarily indicate the absence of a modality effect on the social support–loneliness reduction relationship but rather reflect the limitations inherent in the statistical power of the analysis.

Additionally, it is important to consider other potential confounding variables or moderators that may have influenced the outcomes. Factors such as individual differences in cognitive abilities, sensory impairments, or prior familiarity with technology, though beyond the scope of this study, could potentially modulate the effectiveness of the interventions and, if not adequately controlled for, could confound the results.

In summary, this research makes significant contributions to the literature by providing empirical evidence suggesting that engaging with AI through voice assistant technology can reduce feelings of loneliness and foster a sense of social support among older adults. Furthermore, a discernible modality effect emerges when assessing the efficacy of such technology, demonstrating a preference for the video-based intervention over its audio-only counterpart. This distinction becomes particularly evident when the two outcome variables—loneliness reduction and the enhancement of social support—are evaluated independently.

## 6. Limitations and Future Research

Despite the fact that our study has yielded substantial indications of intervention effects, it is imperative to recognize inherent constraints within this research. Similar to other clinical trials among older adults with a small sample size, our study has the limitations of diminished statistical power and reduced potential for generalizability, particularly in light of the failure to support a subset of our predictions in H6. While the small sample size in our clinical research presents such limitations, it is essential to contextualize this within the broader challenges inherent in data collection among aging adults. Conducting research in this specialized population often means navigating complexities in recruitment and data gathering, which can naturally lead to smaller cohorts. Prospective investigations would benefit from the inclusion of an expanded and more heterogeneous cohort of subjects. Such methodological enhancements would make future studies more accurate in uncovering effects of voice assistant technology amid an increasingly diverse and expanding aging adult demographic.

Future research can look at the relationship between cognition and loneliness, as studies suggest that loneliness can predict dementia [56]. People with different levels of cognitive functioning also may experience modalities of voice assistants differently.

Our findings are consistent with previous studies showing that older adults benefit from multimedia learning by managing cognitive load [57] and enhancing their engagement with technology [58]. However, future investigations that can provide direct evidence of lightened cognitive processing load (e.g., changes in brain activation patterns in functional MRI data) in the V-VAT condition would lend further support to the modality benefits presented in the current report.

Our data were gathered immediately after the relaxation of COVID-19 restrictions across numerous municipalities within the United States. Nevertheless, it is plausible that the ramifications of the COVID-19 pandemic endured for the duration of our investigation. Evidence indicates an augmented utilization and a heightened favorable disposition towards digital technology, including VAT, among older adults during the COVID-19 pandemic [58]. However, the persistence of these trends in a post-pandemic context remains a subject of uncertainty. We advocate for subsequent replications of this research, particularly under varying conditions.

## Figures and Tables

**Table 1:** Descriptive statistics and normality tests of the dependent variables.

Variables	Mean	Median	Variance	Standard Deviation	Range	Skewness	Kurtosis	Shapiro-Wilk Test
Baseline Loneliness	1.85	1.73	0.37	0.61	2.15	0.39, SE = 0.40	−0.77, SE = 0.79	W(34) = 0.96, *p* = 0.266
Post-study Loneliness	1.65	1.58	0.33	0.57	2.60	1.52, SE = 0.40	2.86, SE = 0.79	W(34) = 0.86, *p* < 0.001
Baseline Social Support	5.44	5.54	1.11	1.05	4.33	−0.65, E = 0.40	0.15, SE = 0.79	W(34) = 0.94, *p* = 0.072
Post-study Social Support	5.65	6	1.45	1.20	5.75	−1.58, E = 0.40	3.89, SE = 0.79	W(34) = 0.87, *p* = 0.001

## Data Availability

Data are available upon request.

## Supplementary Materials

The following supporting information can be downloaded at: https://www.mdpi.com/article/10.3390/geriatrics9020022/s1, Instructions for using your Alexa Assistant, Coding schema.
