# Supplementary material for: Reducing Loneliness and Improving Social Support among Older Adults through Different Modalities of Personal Voice Assistants
Source: Geriatrics (Basel). 2024 Feb 22;9(2):22. doi: 10.3390/geriatrics9020022 (PMC10961806; doi:10.3390/geriatrics9020022)
Supplement: Supplementary file 1 [file geriatrics-09-00022-s001.zip › Geriatrics Supplementary Coding schema.pdf]

A Comprehensive Coding Schema for Coding Older People's Voice Interactions with Personal Voice Assistants (PVAs)

I. Overview

- Purpose: To categorize and analyze voice interactions of older people with PVAs.
- Interaction Categories: 12 predefined categories.

II. Predefined Categories

Each interaction is classified into one of the following categories:

1. Morning Greetings: Interactions involving general greetings like "Good Morning," "Morning, Alexa."
2. Afternoon and Evening Greetings: Interactions involving general greetings like "Good Afternoon," "Good Evening," and "Goodnight."
3. Big Sky: Inquiries or commands related to Big Sky, pertaining to detailed weather information.
4. Daily Riddle: Engagements that involve asking for or solving a daily riddle.
5. Five Minute Morning Meditation: Requests or initiations of a brief morning meditation routine.
6. Music: Interactions involving playing music, song requests, or inquiries about musical content.
7. Weather: General weather-related questions or commands, distinct from Big Sky for its broader scope.
8. Asking for a Joke: Requests to hear a joke, typically for entertainment purposes.
9. Playing the Akinator Guessing Game: Interactions related to playing the Akinator game.
10. Calls to Existing Social Connections: Voice commands or queries related to making phone calls to known contacts.
11. Setting Adjustments: Adjustments to the voice assistant's settings, including volume, language, accessibility features, etc.
12. Other Routines: Voice interactions that involve establishing, querying, or executing routines not covered in the above categories.

III. Coding Rules

- Specific Keywords/Phrases within an Uninterrupted Interaction: Identify and use a specific keyword or phrase indicative of each category in an uninterrupted interaction. Repeated keywords or phrases in a single uninterrupted interaction should be counted as one interaction.
- Context Consideration: Understand the context of the interaction to accurately categorize it.
- Multiple Categorizations: If an interaction spans multiple categories, code it under all applicable categories. For example, "Good Morning, Play Music" will be coded as both morning greetings and music categories.
- Review for Ambiguities: Review interactions categorized as "Miscellaneous" for potential inclusion in existing categories.

IV. Data Management

- Recording Method: Utilize a structured format like an EXCEL spreadsheet for data entry.
