# Supplementary material for: Reducing Loneliness and Improving Social Support among Older Adults through Different Modalities of Personal Voice Assistants
Source: Geriatrics (Basel). 2024 Feb 22;9(2):22. doi: 10.3390/geriatrics9020022 (PMC10961806; doi:10.3390/geriatrics9020022)
Supplement: Supplementary file 1 [file geriatrics-09-00022-s001.zip › Geriatrics Supplementary Instructions for Using Alexa Assistant.pdf]

Using your Alexa Assistant

Please do all of the items every day, and make a call once per week

This Echo is a device made by Amazon. It is designed for you to speak to it, ask it questions and give commands. An artificial intelligence (AI) assistant inside of it, called “Alexa,” powers the responses and takes actions at your command.

This list includes a variety of commands for you to use with Alexa, the personal assistant built into your Echo Dot device. Please do all of these every day, and feel free to try your own commands, too. You can just say, “Alexa, what can you do?” and it will share suggestions with you.

To start, just say “Alexa,” and then the Echo device will light up and Alexa will start listening for your request. You must say “Alexa” to make it work. We’ll put a sign that says “Say Alexa” next to the device as a reminder. At any time, tell Alexa to stop what it’s doing by saying “Alexa, stop.” You may need to say “Alexa, stop” before it can start doing something else.

Please remember, the researchers will have access to all the commands and interactions you have with Alexa. If you do not want the researchers to hear one of your Alexa interactions, do not say it to Alexa. Alexa will only record what you say to Alexa, and not other conversations you have in your home.

MORNING COMMANDS: From whenever you wake up until noon

- Say “Alexa, Good morning!”
- Say “Alexa, open Big Sky.” (This will provide detailed weather information)
- Say “Alexa, open Riddle of the Day.”
- Say “Alexa open Five Minute Morning.”
- Say “Alexa, play ___ for 5 minutes”

(Music: Artist/Singer; Band; Album; Style/Genre; Mood/Feeling; Playlist)

Using your Alexa Assistant

Please do all of the items every day, and make a call once per week

AFTERNOON/EVENING COMMANDS: From noon until bedtime

- Say “Alexa, good afternoon!” or “Alexa, good evening!”
- Say “Alexa, what's the weather?"
- Say “Alexa tell me a joke.”
- Say “Alexa, start Akinator.” (This is a trivia-style game)
- Say “Alexa, play ___ for 5 minutes”

(Music; Artist; Singer; Album; Band; Playlist; Style)

CALLING THROUGH ALEXA: Once per week

The researchers will add contact information for special people (friend, family, neighbor) to your Echo device so you can call them weekly. We will also provide instructions to share with them.

- Say “Alexa, call ___ ” (say the person’s name)
- If you have an Echo with a screen, say, “Alexa, video call ______”
